# Public health in the age of longevity interventions: from prevention to system-wide resilience

**DOI:** 10.18632/aging.206381

**Published:** 2026-05-18

**Authors:** Jochen Mierau, Marco Demaria

**Affiliations:** University Medical Center Groningen, University of Groningen, Groningen, the Netherlands; European Research Institute for the Biology of Ageing (ERIBA), Groningen, the Netherlands

**Keywords:** healthy longevity, biological aging, public health, cellular senescence, healthspan

Over the past century, the most transformative gains in human health and longevity did not arise from high-technology medicine, but from the systematic application of public health. Clean water, sanitation, vaccination, safer housing, improved nutrition, poverty reduction, occupational safety, and access to education reshaped population health by preventing disease before it emerged. These interventions reduced early mortality, limited cumulative biological damage, and produced compounding benefits across the life course and across generations. They were scalable, equitable, and preventive by design.

Yet the success of this model has brought us to a fundamentally different challenge. As populations age, the dominant burden of disease is no longer defined by malnutrition, accidents and acute infections, but by chronic conditions, multimorbidity, and progressive loss of function. In many countries, gains in lifespan are no longer matched by gains in healthspan [1]. Individuals are living longer, but with increasing years of disability, frailty, and dependency [2]. This divergence exposes the limits of a system that remains largely organized around the treatment of individual diseases after they arise.

At the same time, the determinants of health have shifted. The foundational public health interventions that drove 20th-century progress remain essential, but they are no longer sufficient to address the systemic exposures that shape aging trajectories in the 21st century. Health is now influenced by a complex interplay of factors that operate continuously across the life course and directly interact with biological aging processes.

Health-harming industries have embedded risk into daily environments through ultra-processed foods, tobacco, alcohol, pollution-related exposures, harmful production practices and others [3]. These risks are often disproportionately affecting vulnerable populations and accelerating the onset of chronic disease. Climate change adds an additional layer of stress, with heat exposure, air pollution, and food system instability contributing not only to acute health events but to long-term physiological decline [4]. Urbanization, sedentary lifestyles, and social disconnection further lead to reduced resilience and increased vulnerability.

In this context, the traditional distinction between prevention and treatment becomes increasingly inadequate. Modern health risks accumulate gradually and manifest across multiple systems, often long before clinical disease is diagnosed. Addressing them therefore requires a more integrated framework that recognizes health as a dynamic trajectory shaped by lifelong exposures, biological responses, and progressive functional decline across the life course.

Such a framework cannot rely exclusively on either conventional public health measures or disease-centered clinical medicine. Instead, healthy longevity will likely depend on the coordinated integration of multiple intervention layers operating at different stages of the disease trajectory. Public health strategies reduce baseline exposure and population vulnerability, clinical medicine manages established pathology, and emerging longevity-directed interventions may help delay or modify the biological processes that connect cumulative damage to disease manifestation and functional decline [5]. Rather than representing separate or competing domains, these approaches should be viewed as complementary components of a unified strategy to improve population health across aging societies.

Importantly, the relevance of longevity-directed interventions is no longer theoretical. A growing body of evidence suggests that modulating core biological pathways, including cellular senescence, inflammation, metabolic dysfunction, and loss of proteostasis, can influence multiple age-related outcomes simultaneously. This creates the possibility of compressing morbidity not by addressing diseases one at a time, but by intervening upstream in the processes that give rise to them [6].

However, realizing this potential will depend not only on scientific progress, but on implementation. The key question is no longer whether longevity interventions can work, but how they can be implemented in an effective and equitable way within existing health systems.

Prevention and health protection is the basis. Interventions that influence environments, from food systems to urban design, from air quality to education, will continue to deliver fundamental advantages, reducing baseline damage and creating conditions of prolonged resilience. Without this, even the most advanced biomedical interventions will have limited impact at the population level.

At the same time, health systems must retain the capacity to respond effectively when disease occurs. Early detection and a timely treatment are essential not only for survival but for preserving function. In aging populations, where multimorbidity has become the norm, coordinated care is a central determinant of healthspan.

It is here that longevity interventions could be introduced and implemented. Rather than replacing prevention or treatment, they should be deployed as part of a coordinated approach that operates across the life course. This requires a shift from viewing these interventions as late-stage therapeutics toward integrating them earlier, at stages where biological decline is detectable but not yet clinically manifest.

There is an important component in timing. Longevity interventions are unlikely to be necessary and/or cost-effective for the entire population at all stages of life. Instead, their greatest impact may lie in individuals or subpopulations at elevated risk of accelerated aging or functional decline. Identifying these groups heavily depends on having the right biomarkers that capture biological rather than chronological age and using them in longitudinal studies.

The model should be based on the following (Figure 1): 1) universal public health measures providing the baseline and targeted preventive strategies addressing high-risk groups; 2) longevity-directed therapies where biological vulnerability becomes evident; and 3) disease-specific treatments once morbidity is already present. Importantly, these layers should not be considered mutually exclusive or strictly sequential. Public health interventions remain essential even after disease onset, while longevity-directed interventions may complement both preventive and disease-specific care by targeting the underlying biological processes that contribute to functional decline and multimorbidity.

**Figure 1:**
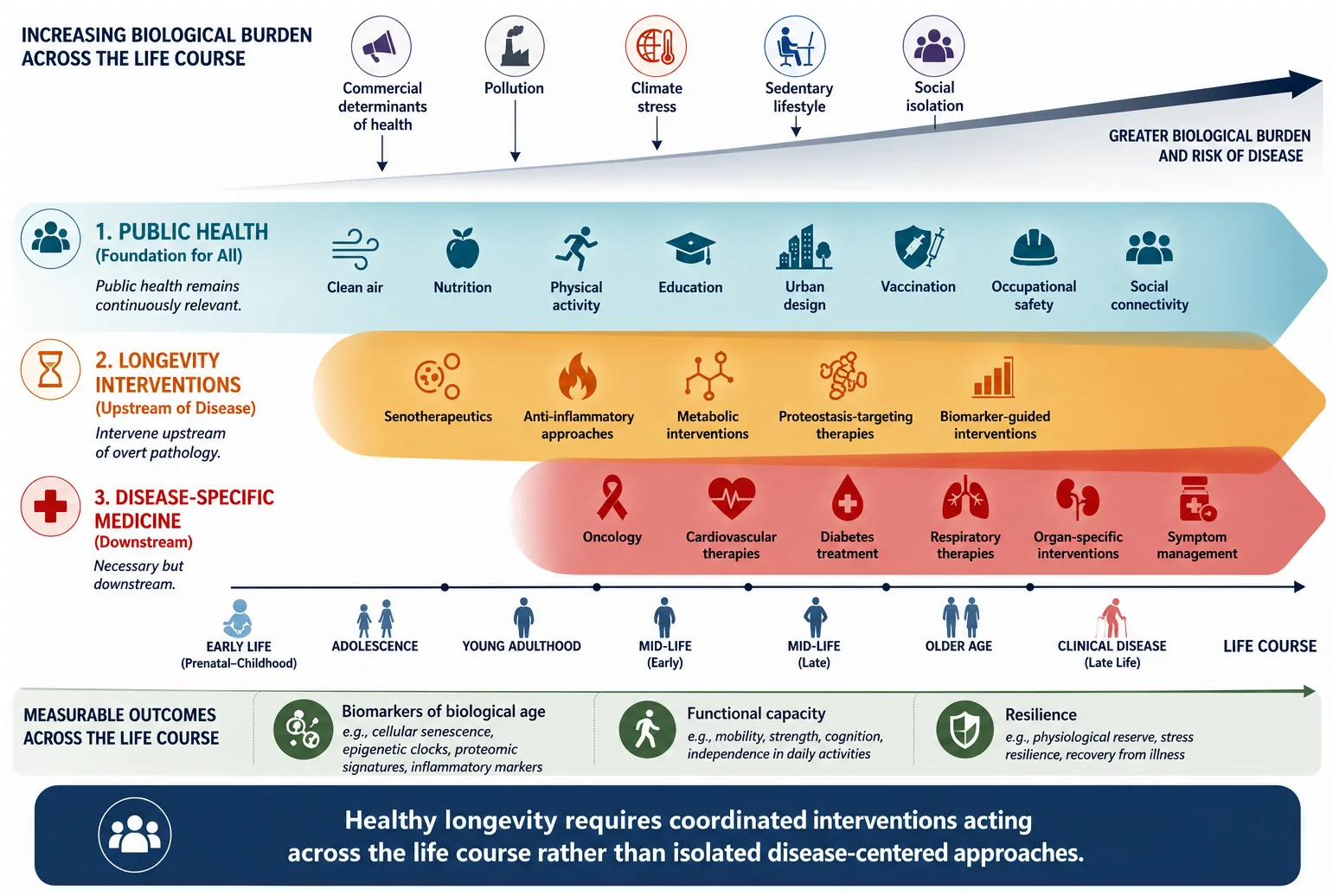
Healthy longevity requires coordinated interventions acting across the life course rather than isolated disease-centered approaches.

A key issue, also from an economic point of view, is that current systems are largely structured around episodic care and disease-specific reimbursement models. These are poorly aligned with interventions that aim to delay or prevent multiple conditions simultaneously. Moving toward prevention-first systems will require new incentive structures that reward long-term outcomes, including functional capacity and independence.

Equally important is the need to measure what matters. Traditional metrics such as mortality and disease incidence capture only part of the picture. For aging populations, outcomes such as physical function, cognitive capacity, resilience, and more generally quality of life are extremely important. Developing robust measures of these outcomes, along with biomarkers of biological aging, is then critical.

There are additional complications for the implementation of such approach. Scaling longevity interventions will require manufacturing capacity, regulatory clarity, clinical infrastructure, and workforce training, in particular for general practitioners. It will also require public trust and clear communication, particularly given the risk of overpromising or misinterpreting early scientific findings. We need to have the right governance, otherwise we risk exacerbating existing inequalities, in particular by guaranteeing access to those who are already advantaged. Moreover, we need to assure that the availability of longevity interventions does not lead to individual and collective reluctance to pursue public health interventions. The history of public health demonstrates that the greatest gains in longevity occur when interventions are widely accessible and distributed equally.

Thus, scientific advances in understanding aging must be matched by policies, financial models, and delivery systems that can translate them into population-level impact. This requires a structured coordination across sectors, including healthcare, public health, urban planning, education, and industry. It also requires a long-term perspective, recognizing that the benefits of prevention and early intervention often accrue over decades rather than years.
